# Preservation of heritage buildings in Alexandria, Egypt: an application of heritage digitisation process phases and new documentation methods

**DOI:** 10.12688/f1000research.123158.2

**Published:** 2023-03-22

**Authors:** Adel El Menshawy, Walid Omar, Sherif El Adawy

**Affiliations:** 1 Department of Architectural Engineering & Environmental Design, Arab Academy for Science Technology & Maritime Transport, Alexandria, Egypt; 2 Department of Architectural, Faculty Fine Arts, Alexandria University, Alexandria, Egypt

**Keywords:** Image-based techniques, Photography, Panoramic photography, Close-range photogrammetry, Heritage Digitisation Process Phases (HDPP), Virtual Reality (VR), Website Heritage Documentation (WHD)

## Abstract

**Background: **Throughout the history of the city, the architecture of Alexandria, Egypt, has been in contact with world cultures, especially those of the Mediterranean sphere. Alexandria is rich with cultural features dating back seven thousand years. In Alexandria, the heritage value of the city has decreased since the beginning of the third millennium of the Common Era because there is no suitable digital documentation system for these more recent assets. The development of a new technique for preserving heritage buildings is required. For example, image- based techniques can gather data using photography, panoramic photography, and close-range photogrammetry.

In this research, we primarily seek to implement Heritage Digitisation Process Phases (HDPP) by introducing both the Building Information Modelling (BIM) environment and the point clouds for achieving a Historic Building Information Modelling (HBIM) model and to establish new documentation methods in architectural conservation and built-heritage preservation, i.e., Virtual Reality (VR) and Website Heritage Documentation (WHD).

**Methods: **The methodology is designed to preserve and manage cultural heritage using HDPP for the promotion of heritage building preservation in Alexandria.

**Results: **The results show that the application of HDPP has led to the creation of a digital database about the Société Immobilière building, which was chosen as a case study for this research.

**Conclusions: **Implementation of HDPP and usage of new documentation methods i.e., VR and WHD create a digital path to help strengthen its image and connect the place to users, recreational areas are created to communicate and explore the city’s architectural history.

## Introduction

Heritage digitisation uses digital media to conserve cultural or natural heritage. In other words, it “consists of unique resources of human knowledge and expression. It embraces cultural, educational, scientific and administrative resources, as well as technical, legal, medical and other kinds of information created digitally, or converted into digital form from existing analogue resources”. ^1^ Moreover, digitisation converts the physical data that pertains to movable or unmovable cultural heritage by using digital technologies to create multidimensional digital archives, which provide architectural data about design theories, building materials, and construction methodologies in different time periods.

Virtual heritage is the result of heritage digitisation. It is focused on recreating tangible cultural heritage by making realistic 3D models depicting them. In other words, it is “the use of computer- based interactive technologies to record, preserve, or recreate artefacts, sites and actors of historic, artistic, religious, and cultural significance and to deliver the results openly to a global audience in such a way as to provide formative educational experiences through electronic manipulations of time and space”. ^2^ Virtualised objects are object models generated from the physical world that surrounds us and the result is placed in a computer’s memory, but the generation process of virtualised objects is different. Hence, virtualised objects or environments result from a measurement process. ^3^

Virtual Reality (VR) is a technology that makes use of computer generated interactive graphics which give the user the sensation of being in a virtual, that is, computer-generated world. ^4^ Virtual heritage is linked to VR, offering a new generation of academics and architects the possibility to use VR technology and tools to create immersive experiences and solutions. Experts have predicted that VR will have an influence on our lives similar to that of the internet or smartphones, with interesting potential in a wide variety of disciplines, including heritage preservation, architecture, education, entertainment, and social action. ^5^

The Heritage Digitisation Process Phases (HDPP) changes documented data that can be perused by individuals to a digital read-only format with the assistance of machines. The digital world is the quickest growing and developing world. Cultural heritage institutions, world heritage sites, libraries, galleries, archives, and museums vary in sort and size across the world, however, within the last decade, the majority of academics and architects use digital technology for digitisation. Current advancements in computer networks, multimedia, VR, and artificial intelligence have given a great establishment to the digitisation of cultural heritage information. The acquisition and perception of cultural heritage information are firmly linked with techniques such as 3D laser scanning or photogrammetry. ^6^ HDPP can be shaped by academics and architects; creating a digital database about heritage buildings that depend on the sort of data (textual data, images, and drawings) that allows users to acquire full knowledge about the documented database and digital library, which features heritage building assets, thus also strengthening users’ attachment to the place.

To start the application of HDPP, there are tools that are to be used for documentation. There are various tools that can be divided into 5 classes (A, B, C, D, and E). Class A is the most expensive, for example, for a terrestrial laser scanner, which is used in interior and exterior works. Matterport Pro2 represents Class B, and Insta360 ONE X2 represents Class D. Matterport Pro2 and Insta360 ONE X2 work by the same concept in capturing; they are almost exclusively used in interiors. Lastly, a DSLR camera represents Class C, and a smartphone represents Class E. DSLR cameras and smartphones will be used in the new method for producing the same results as a terrestrial laser scanner at a low cost and with portability that academics and architects can apply to individual building case studies. The new method will collect data using image-based techniques, which are close-range photogrammetry for specific motifs, photography, and panoramic photography for the entire building’s façades. From the collected data, various platforms will be used to digitally document heritage buildings.

Building Information Modelling (BIM) has evolved significantly in managing and documenting cultural heritage. It can now represent, in a virtual environment, the actual conservation state of the buildings under analysis. However, the virtual reconstruction procedure of historical cultural heritage is difficult because the objects to model consist of components whose heterogeneous, complex, and irregular characteristics and morphologies are not represented in the BIM platform libraries. ^7^ Therefore, it is essential to introduce technical and historical approaches in both the BIM environment and the point clouds, to model the different virtual parametric components and achieve a BIM model for the architectural heritage under analysis. ^8^^,^^9^

Obtaining the parametric building elements from the 3D point cloud is a time-consuming and error-prone manual process, because no automation or platform processes currently exist that can ensure a direct change from the 3D point cloud to full BIM models. ^10^^,^^11^ In this research, we used the 3D point cloud as a reference to build up the BIM model, which took us a period of time to execute. Therefore, once 3D virtual models have been created, the libraries of parametric elements should be generated under Historic Building Information Modelling (HBIM).

Murphy and McGovern developed HBIM to integrate contemporary technology and the BIM approach in cultural heritage documentation. HBIM was first used in 2009. ^12^ HBIM executes the modelling and documenting of architectural elements according to artistic, historical, and constructive typologies. It is designed to preserve and manage cultural heritage using HDPP, which is a four-phase process for the documentation, representation, rendering, and dissemination of heritage building. In addition, HBIM functions as a plug-in for BIM and is considered to be a unique library of BIM parametric objects built from historical data. Furthermore, HBIM serves as a system for mapping the parametric objects onto a 3D point cloud and image survey data. Generally, the HBIM library is built using manuscripts and historical architectural documentation, laser scanning, photogrammetric techniques, and other data obtained from the physical analysis of a building. ^13^

The application of HDPP has led to the creation of a digital database about the Société Immobilière building which was chosen as a case study for this research. The Société Immobilière building represents the features that were prominent in architecture at the time of the building’s construction, which was built when Egypt was a British colony and therefore the building’s design was influenced by European styles.

## Research problem

This research addresses a local and national problem in Egypt that has focused on the decrease in heritage value of certain buildings due to the lack of maintenance and ill-conceived remodeling and additions, which have been built without consideration for the buildings’ original styles. Moreover, there has been no strategy for designating this rich heritage and no suitable system to document these assets. There has also been a shortage in locally authorised officials specialising in photographing, documenting, and using available techniques to capture data about heritage objects, such as 3D laser scanning or photogrammetry. ^14^ One of the major problems is there has been no entity entrusted with keeping this architectural archival documentation or handing it over to the National Organisation for Urban Coordination. Without any methodology to conserve heritage buildings, the city risks further deterioration to its identity and historical value over time. Currently, there is no database to help us better understand design theories through on-site methods that illustrate the historical background, architectural features and styles of a building. Furthermore, documentation methods applied to heritage buildings cannot access the documented asset models and their information.

## Research aim

This research aims to explain the process of documenting heritage buildings by creating a methodology based on digital documentation, architectural conservation, and preservation, in addition to discussing a new documentation technique that provides a digital document about heritage buildings to local architectural heritage organisations. Moreover, this study aims to create a digital database based on technical and historical approaches in both the BIM environment and the point clouds, using video and image survey data, historical drawings and maps of the municipality, plan drawings, and reference books. The digital database helps better understand the relevant design theories through on- site methods, which illustrate the historical background of the heritage buildings and their architectural features and styles. In addition, this research aims to create new documentation methods for heritage buildings that provide access to the documented asset models and their associated information, as well as improve the process of documenting real estate that may be included on the lists of Egyptian Properties Protected from Demolition - Law No. 144 of 2006 and its executive regulations.

## Methods

**Figure 1.**
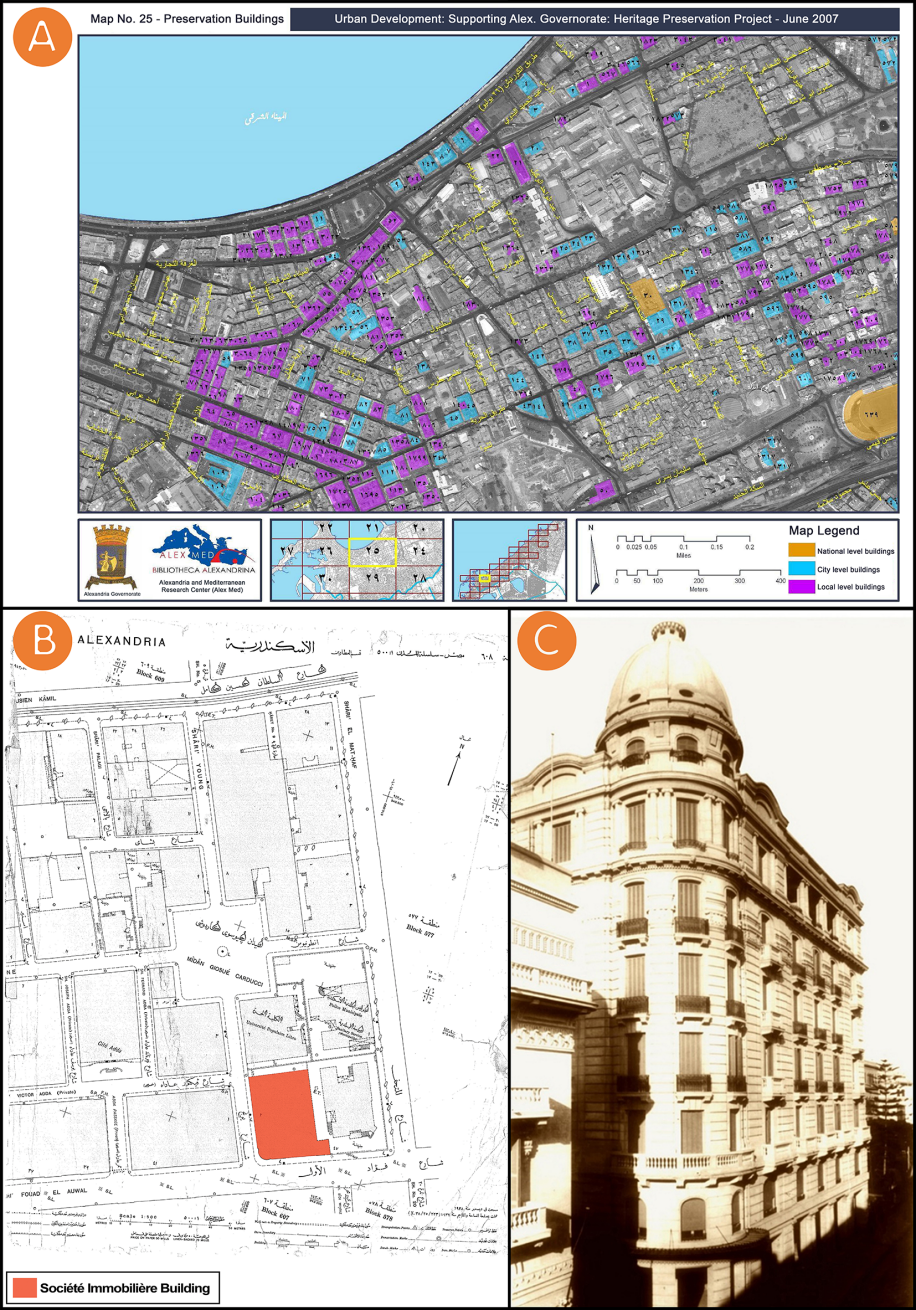
(A) The Société Immobilière building, “item 33/٣٣ in Arabic numbering” is registered under the National Urban Coordination System Volume 2007, the map was acquired from Alex Med of Bibliotheca Alexandrina; (B) Location of Société Immobilière building, 1938, the map was acquired from the City’s Municipal of Alexandria, Egypt; (C) Historic photo of Société Immobilière building, the photo was acquired from Sigma Properties Company. All of (A), (B), and(C) were gathered from open access sources, and available through institutes and their official websites.

The research focuses on heritage buildings in Alexandria, Egypt, and highlights the Société Immobilière building as a case study. The methodology is designed to preserve and manage cultural heritage using HDPP for the promotion of heritage building preservation in Alexandria. The methodology of this study used a method for developing and disseminating heritage digitisation and divides it into four phases. The first phase is that of documenting, which was about finding and analysing information and documenting authentic data emanating from the cultural and architectural past. The authors gathered data, through contact with Alex Med of Bibliotheca Alexandrina and Sigma Properties Company, from reference books, including the “ Conservation and Rehabilitation of Alexandria's City Center” and the “ Patrimoines partagés en Méditerranée” books authored by Dr. Mohamed Awad the founder of “ The Alexandria Preservation Trust”, map and historic photo of Société Immobilière building (see Figure 1-A and Figure 1-C), and general information about the Société Immobilière building ( Table 1). All of these data were gathered from open access sources, and available through institutes and their official websites. In addition, the authors acquired a map of the building location from the City’s Municipal of Alexandria, Egypt (see Figure 1-B), and did image survey data by photography, panoramic photography, and close- range photogrammetry on-site by using DSLR camera with a professional camera tripod, padcaster tripod dolly wheel, smartphone, and DJI Osmo Mobile 3 Gimbal for smartphone. Representation constituted the next phase; after the data has been gathered, they were digitised as a 3D point cloud by using a photogrammetry platform, RealityCapture platform (see Software availability statement for alternatives). Next, the rendering phase produced 3D virtual models by using the BIM platform, Autodesk Revit platform (see Software availability statement for alternative), and also produced realistic images and scenes by using 3D rendering platform, Autodesk Revit and Enscape3D platforms (see Software availability statement for alternatives). The final phase concerned dissemination, which is devoted to the distribution of information and knowledge to the general public through establishing new documentation methods in architectural conservation and built-heritage preservation, i.e., VR and Website Heritage Documentation (WHD). ^15^

**Table 1.** General information about the Société Immobilière building.

Item	Description
Name of the building	Société Immobilière building
Address	52 Fouad Street, Alexandria, Egypt
Year of construction	1928
Structural system	RC skeleton system
Footprint/m ^2^	1,368.88
Total built-up area/m ^2^	11,609.81
Number of façades	Four façades (one on main street, one on side street, and two on back passages)
Number of floors	Basement + ground floor + seven typical floors
Building characteristics	Main and service entrances + two main stairwells + central court and service stairs
Current owned by	Abdel Meguid Mostafa and Partners (established in 1959)
Developer/Manager	Sigma Properties Company (established in 2008)

### Case study: The Société Immobilière building, Alexandria, Egypt

The city of Alexandria was founded by Alexander the Great and early on featured a fusion of communities – Muslims, Christians, Jews, Armenians, Greeks, and Italians. It was once considered the jewel of the Mediterranean. Within the city, a case study was selected among sites with remarkable character. The selected case study is visible from the main roads in the area and represents a heritage style that has been understudied in the literature. Located at 52 Fouad Street, Alexandria, Egypt, the Société Immobilière building was chosen as a case study for this research (see Figure 1-A and Figure 1-B). Fouad Street is one of Alexandria’s oldest streets and the most powerful symbol of Alexandria’s remarkable history, with its elegant villas and antique shops. The Société Immobilière building is one of the Sigma Properties Company’s most famous acquisitions. Indeed, the Sigma Properties Company saw the Société Immobilière building as an excellent investment opportunity. Designed in a unique Neo-Renaissance architectural style, the property has been listed as “item 33” on the Heritage List and registered in the National Urban Coordination System Volume 2007 (see Figure 1-A). ^16^^,^^17^

The Greek architects N. Paraskevas and P. Gripari designed the building. The Italian Averino family hired them after problems had emerged with their previous architect Giacomo Loria. ^18^^,^^19^ The eclectic revivalist style of the late nineteenth century expressed perfectly the architectural pluralism and pro- European cosmopolitanism that prevailed in the city at the time. Later, between the two world wars, the decorative and early modern international styles became the most favoured architectural expressions in the city. ^19^^,^^20^ The building under study was designed according to the popular eclectic aesthetics of the Neo- Renaissance style, and the building boasts a distinctive dome that can be seen from a distance (see Figure 1-C). The building has been used as a meeting chamber for Alexandria’s elites and still stands out among many residences as a testimony to the wealth and pluralism of the city’s cosmopolitan identity. The following table ( Table 1) summarises general information about the Société Immobilière building. ^19^^,^^21^

This study developed an application that seeks to digitally document one of Alexandria’s most distinguished architectural heritage assets, exemplifying the rich architectural heritage found in Egypt. This application of HDPP (documentation phase, representation phase, rendering phase, and dissemination phase) creates a digital library to document heritage buildings’ assets using the best suited available techniques to introduce an interactive 3D representation of the entire building’s façades and to model the entire building’s façades.

### Documentation phase

**Figure 2.**
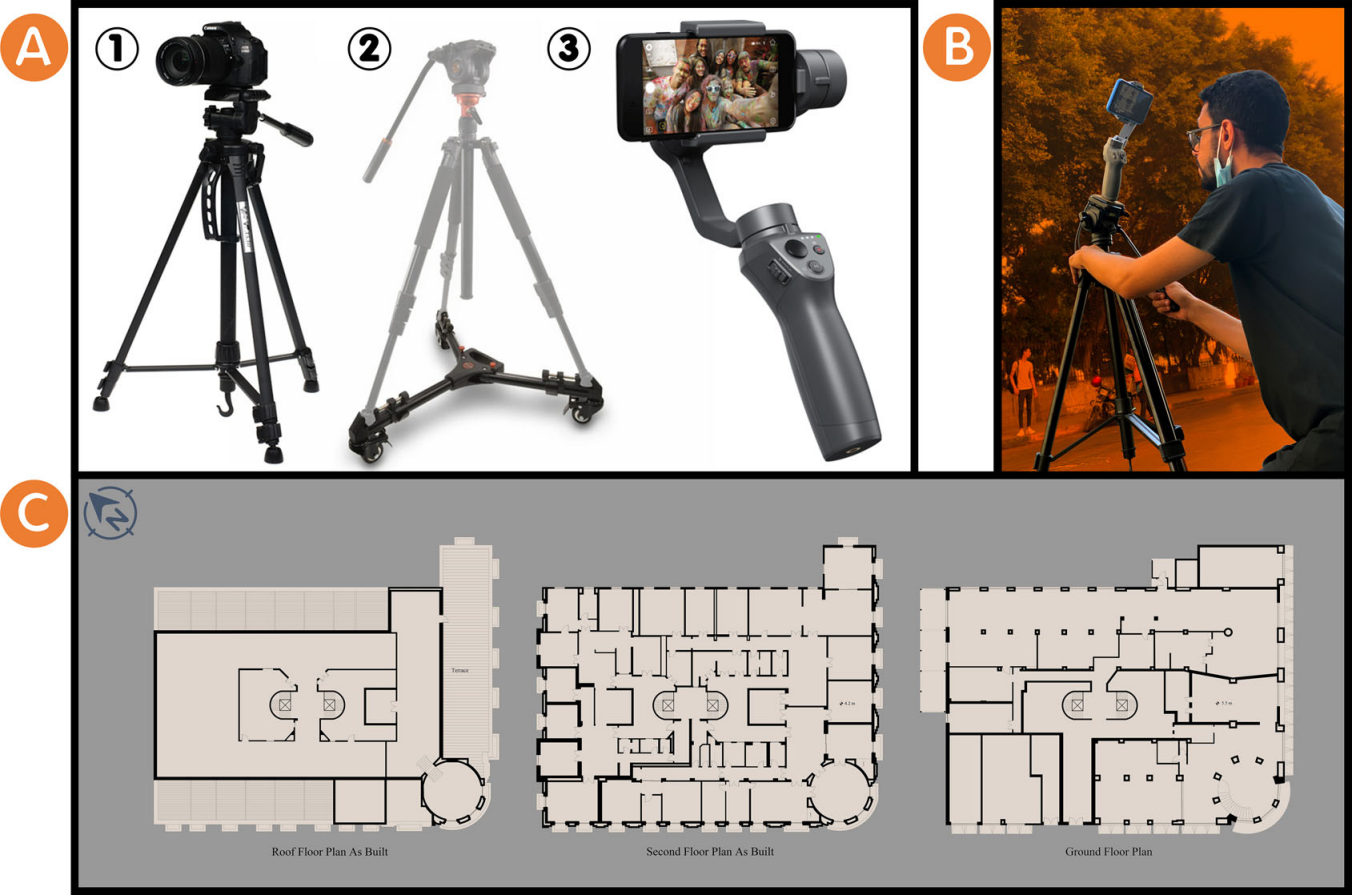
(A) The tools used in surveying the Société Immobilière building are (1) DSLR camera with a professional camera tripod, (2) Padcaster tripod dolly wheel, and (3) Smartphone with DJI Osmo Mobile 3 Gimbal for smartphone, the photos were acquired from Google Images; (B) One of the researchers is surveying the Société Immobilière building using photography and panoramic photography and close-range photogrammetry on-site, the photo was acquired from the researchers; (C) Plan drawings of Société Immobilière building, the drawings were acquired from the researchers’ survey and draw the Société Immobilière building indoors and outdoors.

Image-based techniques were used to gather data using photography and panoramic photography for the entire building’s façades and close-range photogrammetry for specific motifs. The tools used are as follows (see Figure 2-A):

• DSLR camera and smartphone.
• Professional camera tripod.
• Padcaster tripod dolly wheels.
• DJI Osmo Mobile 3 Gimbal for the smartphone.

Other sources of information were used, where researchers contacted Alex Med of Bibliotheca Alexandrina, Sigma Properties Company, and the City’s Municipal of Alexandria, Egypt, to gather data for the Société Immobilière building under the regulations of registration in the National Urban Coordination System (see Figure 1-A), its location map in 1938 (see Figure 1-B), and historic photo for the building (see Figure 1-C) as reference documents for it. Moreover, the researchers surveyed and drew the Société Immobilière building indoors and outdoors as plan drawings of it (see Figure 2-B and Figure 2-C). All the above served to obtain information about the building from the time of its construction to the present day. The following table ( Table 2) summarises the documentation phase for the Société Immobilière building.

**Table 2.** Documentation phase for the Société Immobilière building.

Item	Description
Heritage type	Tangible cultural heritage – Mixed use
Grading criteria for listed heritage buildings	Grade 2
Grading system of heritage buildings	Grade B
Levels of intervention for conservation of heritage buildings	Level two (Rehabilitation)
Architectural styles	Neo-Renaissance architecture
Documentation techniques	Image-based techniques
Tools	DSLR camera and smartphone
Output data	Photography, panoramic photography, and close-range photogrammetry

### Representation phase

**Figure 3.**
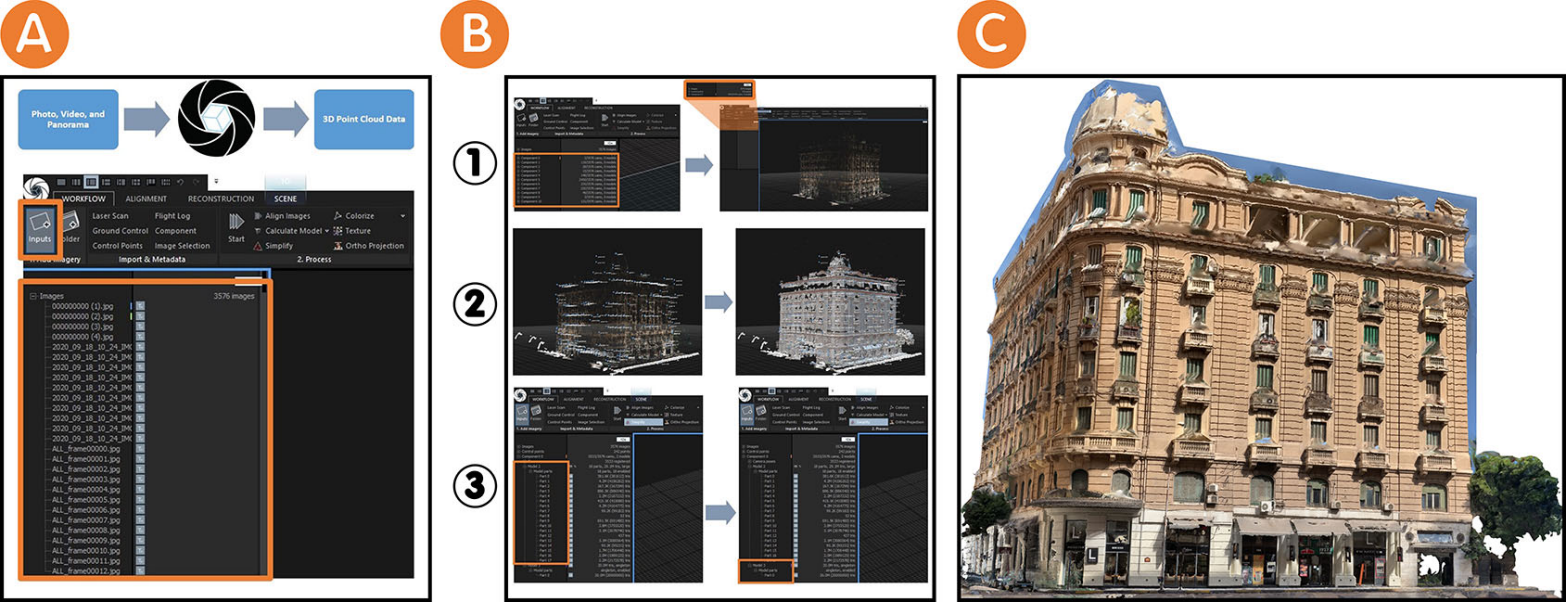
(A) Importing 2D data to the RealityCapture platform and creating 3D point cloud data as the output of 2D data; (B-1) Aligning images processed through the RealityCapture platform to form 3D point cloud data as one component, (B-2) Reconstruction process through the RealityCapture platform to form one component as a mesh model, and (B-3) Simplification process through the RealityCapture platform to optimise the mesh model; (C) Texturing process through the RealityCapture platform to create a detailed 3D textured map and coloured triangle mesh. The photos (A), (B), and(C) were acquired from the researchers’ working on the Société Immobilière building by using RealityCapture platform.

After the data have been gathered, they were digitised as a 3D point cloud. This 3D point cloud represents the data about the Société Immobilière building’s façades that was obtained during the documentation phase. Next, the representation phase using a photogrammetry platform follows these sequential steps:

• Photos, videos, and panoramas were imported (3576 images) as 2D data into the RealityCapture platform (see Figure 3-A). Images were aligned in order to align 2D data and register the images. This process ended with the creation of 3D point cloud data in the form of one single component.
• The alignment of image processing may be duplicated because it may not produce one single component upon the first trial. This process led researchers to create “control points” as 1D data on the 2D data to add more detailed information. This process was duplicated until the 3D point cloud data become a single component (see Figure 3-B-1).
• The reconstruction process means that the component is reconstructed as a mesh model using graphics hardware. At this point, we had two options: a “normal detail” or a “high detail” to reconstruct the component. In this study, the researchers choose the “high detail” option, since it gives a more accurate meshing details, although it requires a longer time (see Figure 3-B-2).
• The simplification process is the process by which the model’s mesh size is optimised by reducing the triangle count. Once the researchers in this study were satisfied with the appearance of their reconstruction, it is good practice to define the overall triangle target for the 3D experiences. This practice represents the fidelity researchers seek to achieve (see Figure 3-B-3).
• The texturing process uses source images to create a detailed 3D textured map and coloured triangle mesh (see Figure 3-C). The export mesh process can then export the project in many different file formats. We exported as an OBJ file for this workflow. OBJ file is a safe and standard format, although FBX file and GLB file are also good options.

The following table ( Table 3) summarises the representation phase for the Société Immobilière building.

**Table 3.** Representation phase for the Société Immobilière building.

Item	Description
Representation platforms	RealityCapture
Input data	Photos, videos, and panoramas
Output data	3D point cloud data and 3D textured and coloured triangle mesh

### Rendering phase

**Figure 4.**
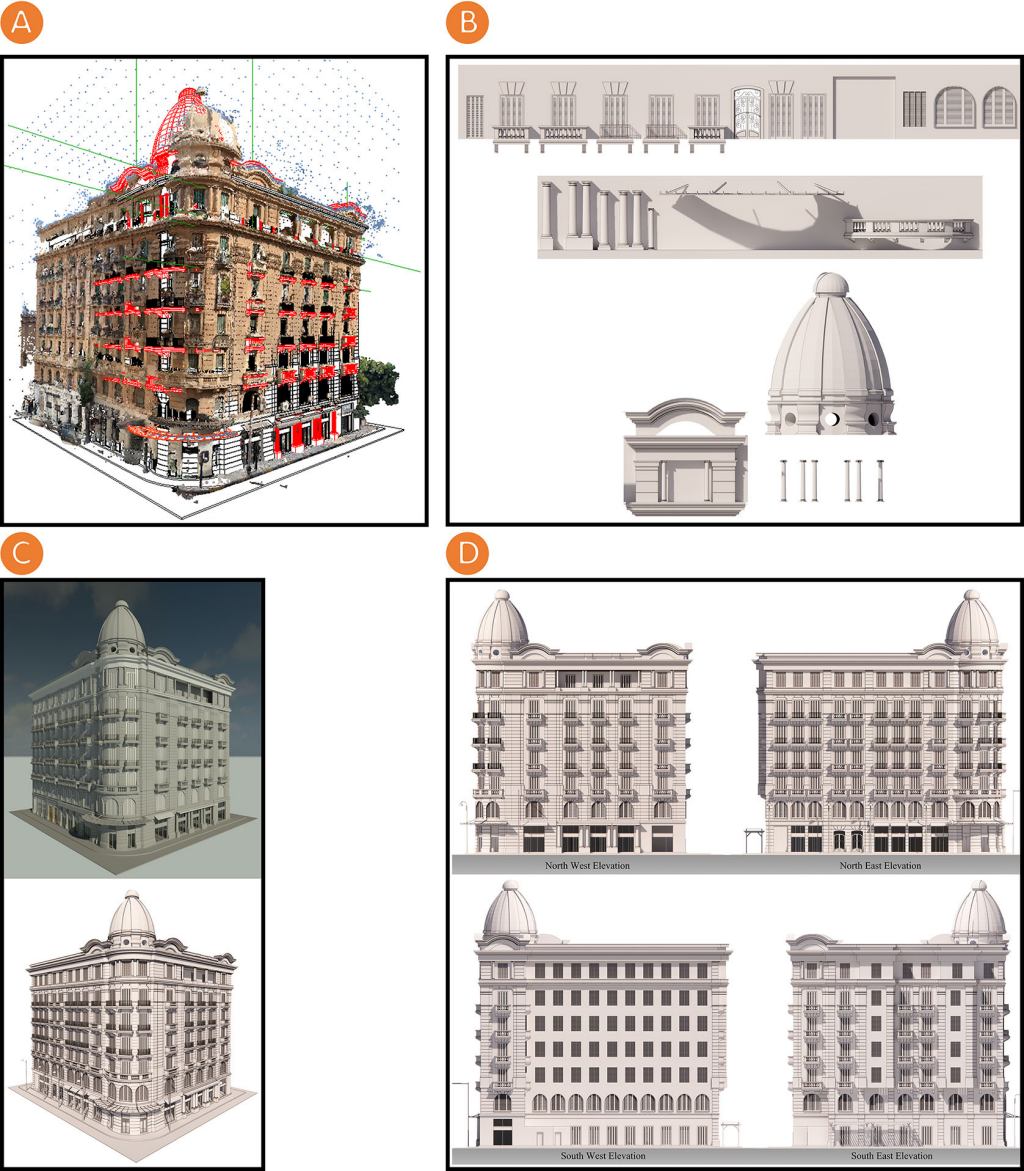
(A) Importing 3D textured and coloured triangle mesh on Autodesk Revit platform for the documentation and modelling of Société Immobilière building; (B) Creating a RFA, which is a unique library of BIM parametric objects created by the researchers on Autodesk Revit platform used on documentation and modelling of Société Immobilière building; (C) Top - Rendering the Société Immobilière building on Autodesk Revit platform, Bottom - Rendering the Société Immobilière building on Enscape3D platform; (D) Top - North-west and north-east elevations of the Société Immobilière building rendered by using Enscape3D platform, Bottom - South-west and south-east elevations of the Société Immobilière building rendered by using Enscape3D platform. The photos (A), (B), (C), and (D) were acquired from the researchers’ working on the Société Immobilière building by using Autodesk Revit and Enscape3D platforms.

The rendering phase using BIM and 3D rendering platforms follows these sequential steps:

• 3D textured and coloured triangle mesh is imported on the Autodesk Revit platform to document and model the Société Immobilière building’s façades (see Figure 4-A).
• A Revit Family (RFA) is created. It is a unique library of BIM parametric objects (templates and 3D models of various objects i.e., doors, windows, stairs and so on) created by researchers on the Autodesk Revit platform, and it is used to document and model the Société Immobilière building’s façades (see Figure 4-B).
• The Société Immobilière building is rendered on Autodesk Revit and Enscape3D platforms (see Figure 4-C).

The documenting and modelling of the Société Immobilière building’s façades were accomplished by using the Autodesk Revit platform and creating an RFA, a unique library of BIM parametric objects. During the rendering phase, we create a visualisation environment for the Société Immobilière building by visualising the resulting documentation model that illustrates the Société Immobilière building’s architectural style. Figure 4-D shows the rendered elevations using the Enscape3D platform which are the researchers’ work of this study; these elevations represent output to document the Société Immobilière building. This phase culminated with the creation of an HBIM model, offering a database and digital library about the Société Immobilière building’s assets.

The following table ( Table 4) summarises the rendering phase for the Société Immobilière building.

**Table 4.** Rendering phase for the Société Immobilière building.

Item	Description
Rendering methods	Autodesk Revit and Enscape3D
Input data	3D textured and coloured triangle mesh
Output data	HBIM model with database and digital library of documented heritage building’s assets

### Dissemination phase

We created new documentation methods for the Société Immobilière building. The VR and WHD both provide access to the documented HBIM model and its associated information and improve the process of documenting real estate intended for the real estate inventory lists of Egyptian Properties Protected from Demolition - Law No. 144 of 2006 ^22^ and its executive regulations. These two methods allow users to fully know the documented database and digital library that include information about the assets of documented heritage buildings. Furthermore, the Société Immobilière building is preserved over time based on its HBIM model and digital technologies.

The dissemination phase constitutes the final phase in the HDPP and entails the dissemination of information about heritage to the public. As part of our case study, we created two methods to disseminate digital documentation about the Société Immobilière building.

**Figure 5.**
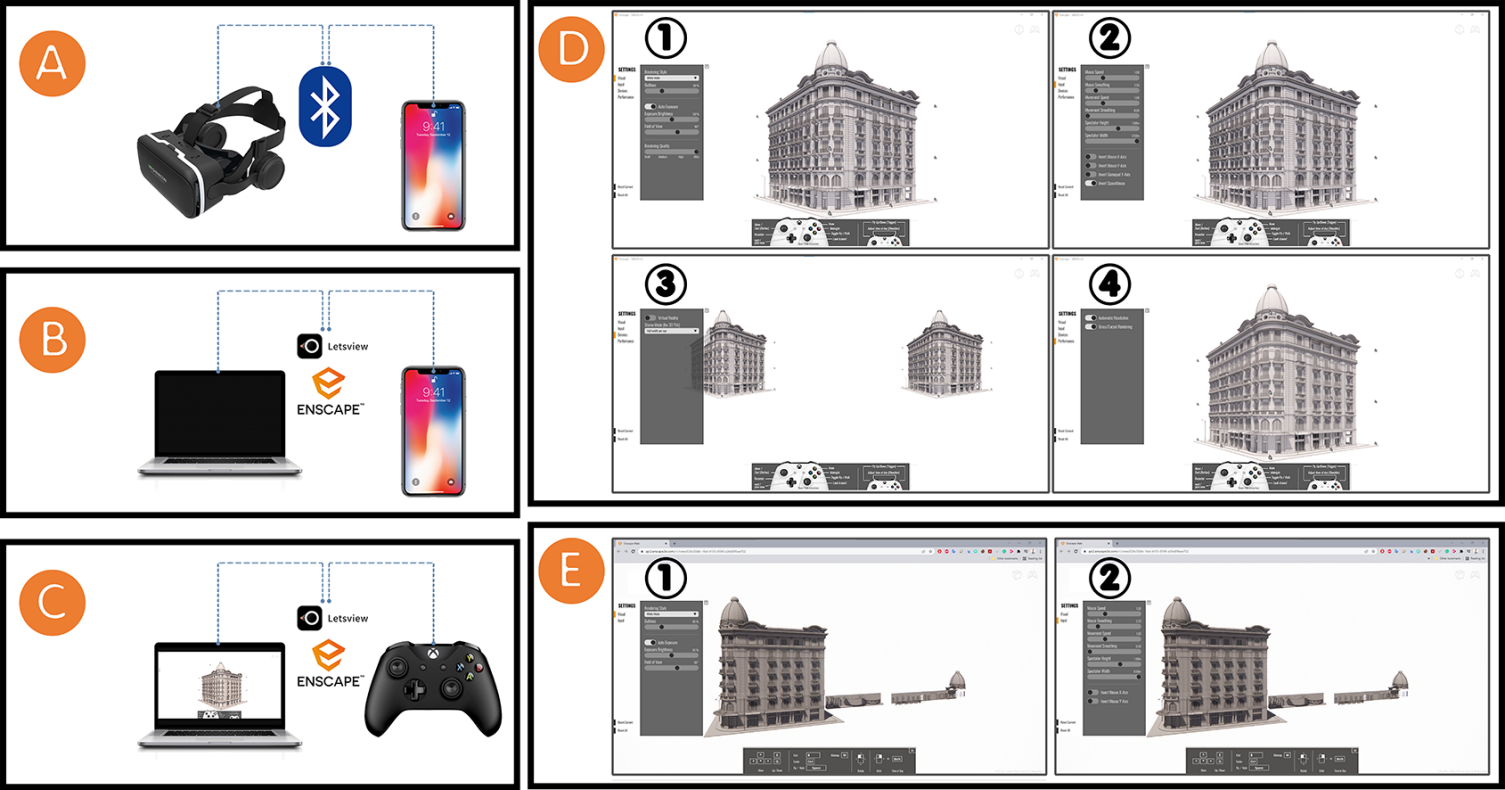
(A) Installing and connecting a smartphone to a Shinecon VR headset SC-G04 through Bluetooth; (B) Mirroring the stream of HBIM model from a PC/laptop to a smartphone through the Letsview platform; (C) Navigating the HBIM model by using Microsoft Xbox One Wireless Controller; (D) Modifying the VR settings for the Société Immobilière building (1) “Visual,” (2) “Input,” (3) “Devices,” and (4) “Performance” sub-tabs; (E) Modifying the settings for the Société Immobilière building’s website (1) “Visual,” and (2) “Input” sub-tabs. The photos (A), (B), (C), (D), and (E) were acquired from the researchers’ working on the Société Immobilière building by using Enscape3D platform.

We first created a VR for the Société Immobilière building. This VR experience uses the placement of the HBIM model to build a scene within the Enscape3D platform, integrating motion control and interactions in the Enscape3D platform. Finally, the immersive and interactive visualisation of the Société Immobilière building was integrated in the VR system. The setup for the VR system was as follows:

• We installed a smartphone in a VR headset ( Shinecon VR headset SC-G04, used in this research) and connected the headphones with a smartphone through Bluetooth (see Figure 5-A).
• We mirrored the stream of the HBIM model in Real-Time (RT) rendering on the Enscape3D platform from a PC/laptop to a smartphone through the Letsview platform, a free wireless screen mirroring platform (see Figure 5-B).
• We used a wireless controller ( Microsoft Xbox One Wireless Controller, used in this research) to navigate the HBIM model in RT rendering on the Enscape3D platform (see Figure 5-C).
• We modified the settings in the VR for the Société Immobilière building by entering the “settings” tab and changing the “visual,” “input,” “devices,” and “performance” sub-tabs (see Figure 5-D).

Our second method was to create a website for the Société Immobilière building, by using the placement of the HBIM model to build a scene within the web version of the Enscape3D platform, to integrate motion control and interactions in the web version of the Enscape3D platform, and finally, to create an immersive and interactive visualisation of the Société Immobilière building in RT rendering. The settings for the Société Immobilière building’s website can be modified by clicking on the “settings” tab and modifying the “visual” and “input” sub-tabs (see Figure 5-E).

The following table ( Table 5) summarises the dissemination phase in the Société Immobilière building study.

**Table 5.** Dissemination phase for the Société Immobilière building.

Item	Description
Dissemination methods	Enscape3D
Input data	HBIM model
Output data	Virtual Reality and Website Heritage Documentation

## Results and discussion

In this research, we created new documentation methods and applied them to the Société Immobilière building. In the following points, they are summarizing the phases' timeline for implementing the HDPP on the Société Immobilière building:

• Documentation Phase: We collected data using image-based techniques, which are close-range photogrammetry for specific motifs, photography, and panoramic photography for the entire building’s façades. It took four days because we faced difficulties in taking stabilised photos and in collecting the photos. We overcame these challenges by using a professional camera tripod, padcaster tripod dolly wheels, and the DJI Osmo Mobile 3 Gimbal for the smartphone to stabilize photos taken of building façades in grid form by dividing the façades into vertical and horizontal portions. Moreover, we surveyed and drew the Société Immobilière Building indoors and outdoors as planned, which took three days.
• Representation Phase: For four weeks, we tested various photogrammetry platforms. We faced errors and difficulties in merging the pictures, defining the façade details, and building up the mesh of the model. Finally, whenever we used the RealityCapture platform, which was powerful and stable with good compatibility with the BIM platforms, we finished our work on it. Throughout the fourteen hours we used the RealityCapture platform, we achieved many processes, including alignment, depth-map computation, meshing, post-processing, colouring, unwrapping, simplifying, and texturing processing time.
• Rendering Phase: For four months, we documented and modelled the Société Immobilière Building as well as created RFA, a unique library of BIM parametric objects on the Autodesk Revit platform. We rendered on the Enscape3D platform which took one minute.
• Dissemination Phase: “VR for the Société Immobilière Building” and “Website for the Société Immobilière Building” which took between 1–2 minutes.

Based on the Société Immobilière building, VR and WHD were used to provide access to the documented HBIM model and its corollary information and improve the process of documenting real estate intended for the inventory lists of Egyptian Properties Protected from Demolition - Law No. 144 of 2006 and its executive regulations. These two methods through the application of HDPP lead to the creation of a digital database about the Société Immobilière building that allows users to acquire full knowledge about the documented database and digital library, which feature heritage building assets, thus also strengthening users’ attachment to the place. Furthermore, the Société Immobilière building is revitalized over time based on the HBIM model and digital technologies (RealityCapture, Autodesk Revit, and Enscape3D platforms). The database result from the application tends to digitalise heritage buildings in 3D format and create digital libraries for those buildings. These new documentation methods open new possibilities for the documentation of heritage buildings and for new strategies for the preservation of Alexandria’s architectural styles. Furthermore, academics and architects can deepen their knowledge about different design theories through the dissemination of these new documentation methods.

## Conclusions

The research uses the implementation of HDPP and the usage of new documentation methods to communicate with architectural heritage data using image-based techniques accompanied by BIM, photogrammetry, and 3D rendering platforms to document heritage that tends to have a suitable digital documentation system for heritage buildings’ assets. HDPP facilitates architectural research by creating a digital database based on video and image survey data, historical drawings and municipal maps, plan drawings, and reference books. The digital database helps better understand design theories through the on-site methods that highlight the history, architectural features, and styles of buildings, which increases the value of the heritage buildings.

The documentation methods, i.e., VR and WHD, enhance the availability of modelling and advanced curricular resources, allowing for global participation in the dissemination of digital documentation about heritage buildings.

This research uses the Société Immobilière building as a case study that was a starting point for applying new documentation methods in Alexandria, Egypt. Our methods document this building and create a digital path to help strengthen its image and connect the place to users, recreational areas are created to communicate and explore the city’s architectural history. In addition, these documentation methods facilitate the preservation of heritage buildings using digital technologies and serve as a digital academic reference for the heritage buildings of Alexandria, Egypt.

The advantages of the researchers’ experience from this research are that we used DSLR cameras and smartphones to produce the same results as a terrestrial laser scanner at a low cost, and that we used the RealityCapture platform, which was powerful and stable with good compatibility with the BIM platforms. The disadvantages were the specification and cost of a powerful workstation needed to deal with the application of HDPP, and we recommend having a Servers Farm to make the application easier to use and to provide better functionality and accessibility in order to adapt to larger project scales in the future. Another disadvantage was the time it took in the documentation phase, for which we recommend using a terrestrial laser scanner if it is available; this would take less time in the documentation phase.

## Data availability

Mendeley: HDPP, Société Immobilière building, Alexandria, Egypt, https://data.mendeley.com/datasets/jy43tptxzz/2. ^23^

This project contains the following underlying data:

• RC (1).png (A figure shows aligning images processed through the RealityCapture platform to form 3D point cloud data as one component).
• RC (2).png (A figure shows the reconstruction process through the RealityCapture platform to form one component as a mesh model).
• RC (3).png (A figure shows texturing process through the RealityCapture platform to create a detailed 3D textured map and coloured triangle mesh – 01).
• RC (4).png (A figure shows texturing process through the RealityCapture platform to create a detailed 3D textured map and coloured triangle mesh – 02).
• REVIT (1).png (A figure shows a rendering of the Société Immobilière Building on the Autodesk Revit platform – 01).
• REVIT (2).png (A figure shows a rendering of the Société Immobilière Building on the Autodesk Revit platform – 02).
• REVIT_RC (1).png (A figure shows importing 3D textured and coloured triangle mesh on Autodesk Revit platform to documentation and modelling of Société Immobilière Building – 01).
• REVIT_RC (2)-VocXt9.png (A figure shows importing 3D textured and coloured triangle mesh on Autodesk Revit platform to documentation and modelling of Société Immobilière Building – 02).
• VR01.png (A figure shows modifying the VR settings on the Enscape3D platform for the Société Immobilière Building – Visual sub-tab).
• VR02.png (A figure shows modifying the VR settings on the Enscape3D platform for the Société Immobilière Building – Input sub-tab).
• VR03.png (A figure shows modifying the VR settings on the Enscape3D platform for the Société Immobilière Building – Devices sub-tab).
• VR04.png (A figure shows modifying the VR settings on the Enscape3D platform for the Société Immobilière Building – Performance sub-tab).
• WEB01.png (A figure shows modifying the settings for the Société Immobilière Building on the web version of the Enscape3D platform – Visual sub-tab).
• WEB02.png (A figure shows modifying the settings for the Société Immobilière Building on the web version of the Enscape3D platform – Input sub-tab).
• XBOX E.png (A figure shows navigating the HBIM model on the Enscape3D platform by using Microsoft Xbox One Wireless Controller).

Data are available under the terms of the Creative Commons Attribution 4.0 International license (CC-BY 4.0).

## Software availability

• RealityCapture free alternatives: COLMAP and Meshroom platforms.
• Autodesk Revit free alternative: TAD platform.
• Autodesk Revit and Enscape3D free alternatives: Blender and LuxCoreRender platforms.
