# Supplementary material for: Preservation of heritage buildings in Alexandria, Egypt: an application of heritage digitisation process phases and new documentation methods
Source: F1000Res. 2023 Mar 22;11:1044. Originally published 2022 Sep 13. [Version 2] doi: 10.12688/f1000research.123158.2 (PMC10043631; doi:10.12688/f1000research.123158.2)
Supplement: Peer review history

# Peer review: Reviewer response for version 2

**DOI:** 10.5256/f1000research.145905.r167336

**Published:** 2023-03-27

**Authors:** Osama Omar

**Affiliations:** 1 Faculty of Architecture‐Design and Built Environment, Beirut Arab University, Beirut, Lebanon

Dear Authors,

 I would like to thank the authors for their effort.

 The authors have made significant efforts to develop and implement Heritage Digitisation Process Phases (HDPP). Moreover, the authors establish new documentation methods in architectural conservation and built-heritage preservation, through using Virtual Reality (VR) and Website Heritage Documentation (WHD)

 After careful revision, kindly know that authors respond to each comment that I given to them. For that I Approve the paper in its current version of submission.

 By the way, I want to say that you did a great job.

 Thank you for your efforts.

 Best Wishes

Is the work clearly and accurately presented and does it cite the current literature?

Yes

If applicable, is the statistical analysis and its interpretation appropriate?

Yes

Are all the source data underlying the results available to ensure full reproducibility?

Yes

Is the study design appropriate and is the work technically sound?

Yes

Are the conclusions drawn adequately supported by the results?

Partly

Are sufficient details of methods and analysis provided to allow replication by others?

Yes

Reviewer Expertise:

BIM, Intelligent Buildings, Zero Energy, Smart Cities, Daylighting

I confirm that I have read this submission and believe that I have an appropriate level of expertise to confirm that it is of an acceptable scientific standard.

# Peer review: Reviewer response for version 1

**DOI:** 10.5256/f1000research.135235.r152650

**Published:** 2022-10-13

**Authors:** Nabil Ibrahim Mohareb

**Affiliations:** 1 Department of Architecture, The American University in Cairo, Cairo, Egypt

The paper offers an interesting digital archiving method for one of Alexandria's well-known buildings. The paper is well organized. My comments are a mix of technical and general thoughts.

- It is preferable to define the designated era in the abstract section, as it is not evident in the text ‘... there is no suitable documentation system for these more recent assets.’ 
- In the introduction, it was difficult to spot the research aim directly. I think this is the main focus/aim of the research ‘... Therefore, it is essential to introduce technical and historical approaches in both the BIM environment and the point clouds, to model the different virtual parametric components and achieve a BIM model for the architectural heritage under analysis.’ I would strongly advise you to emphasize this in both the abstract and the introductory sections.
- In the literature review section, other photogrammetry and digital archiving strategies (methods/approaches) could be compared (briefly) with the recommended methods and tools to demonstrate that your suggested tools are more efficient.
- ‘... Obtaining the parametric building elements from the 3D point cloud is a time-consuming and error-prone manual process because no automation or platform processes currently exist that can ensure a direct change from the 3D point cloud to full BIM models.’ If this is the main research gap, it is preferable to indicate so explicitly in the introduction.
- Your readers may benefit from a definition of technical phrases such as 3D point cloud.
- There is no mention of ground sampling distance (GSD). It is essential to understand how your model gets scaled. When photogrammetric techniques are utilized, metric data cannot be disregarded. Please elaborate on technical concerns pertaining to the camera and image information.
- It would be informative to specify whether the final 3D model reflects only the building's two façades or the entire building.
- In the conclusion section, I would propose highlighting any potential limits of this technique and comparing it to other methods (refer to my earlier comment) to highlight its advantages and disadvantages.

Is the work clearly and accurately presented and does it cite the current literature?

Partly

If applicable, is the statistical analysis and its interpretation appropriate?

Not applicable

Are all the source data underlying the results available to ensure full reproducibility?

Yes

Is the study design appropriate and is the work technically sound?

Yes

Are the conclusions drawn adequately supported by the results?

Yes

Are sufficient details of methods and analysis provided to allow replication by others?

Yes

Reviewer Expertise:

Spatial configuration analysis for historical cities; photogrammetry; movement analysis.

I confirm that I have read this submission and believe that I have an appropriate level of expertise to confirm that it is of an acceptable scientific standard.

# Response

**Published:** 2023-03-20

**Authors:** Sherif El Adawy

**Affiliations:** Arab Academy for Science Technology & Maritime Transport, Egypt

We would like to thank you, Dr. Nabil Ibrahim Mohareb, for your comments.

 In replying to your comments, we took your comments into consideration for the new version of the article:

1. We defined the designated era in the abstract section: “In Alexandria, the heritage value of the city has decreased **since the beginning of the third millennium of the Common Era** because there is no suitable **digital** documentation system for these more recent assets.”
2. We explained the aim of the research in the abstract: “In this research, we primarily seek to implement Heritage Digitisation Process Phases (HDPP) **by introducing both the Building Information Modelling (BIM) environment and the point clouds for achieving a Historic Building Information Modelling (HBIM) model** and **to** establish new documentation methods in architectural conservation and built-heritage preservation, i.e., Virtual Reality (VR) and Website Heritage Documentation (WHD).” And in “Research aim” section, we  clarified the creation of the digital database was **based on technical and historical approaches in both the BIM environment and the point clouds, using video and image survey data, historical drawings and maps of the municipality, plan drawings, and reference books**.
3. We mentioned the tools related to photogrammetry and digital archiving and selected the tools we used in the case study.
4. Throughout these words, "Obtaining the parametric building elements from the 3D point cloud is a time-consuming and error-prone manual process because no automation or platform processes currently exist that can ensure a direct change from the 3D point cloud to full BIM models", we wanted to figure out that there is no direct platform to convert the 3D point cloud to a BIM model, as well as that **we took the 3D point cloud as a reference to build up the BIM model, which took us a period of time to execute**. Referring to the main research gap, this was not the main research gap for this article, but we would consider this research gap in our consideration for our next article. 
5. All technical terms, such as the 3D point cloud, have been hyperlinked, which was recommended by the editor to help readers find up-to-date information on terminologies, organizations, platforms, laws, books, people, and tools.
6. In terms of ground sampling distance (GSD), we did not use a drone as a photogrammetric technique in our article, instead employing a new method that included a DSLR camera, a smartphone, a professional camera tripod, a padcaster tripod dolly wheels, and a DJI Osmo Mobile 3 Gimbal for the smartphone.
7. We indicated that the final 3D model is for the entire building’s façades by adding the word " **entire**."
8. We have covered the advantages and disadvantages of what we got out of our experiences in the conclusion section.

# Peer review: Reviewer response for version 1

**DOI:** 10.5256/f1000research.135235.r151718

**Published:** 2022-10-04

**Authors:** Osama Omar

**Affiliations:** 1 Faculty of Architecture‐Design and Built Environment, Beirut Arab University, Beirut, Lebanon

Dear Authors,

 I would like to thank the author/s for their effort.

 A significant effort has been made by the authors to develop and implement Heritage Digitisation Process Phases (HDPP). Moreover, the authors establish new documentation methods in architectural conservation and built-heritage preservation, through using Virtual Reality (VR) and Website Heritage Documentation (WHD). But there are some minor comments that need from authors to modify it to enhance the research:

1. In the Introduction: it`s so brief and short. So, I prefer to expand the references in this area especially in Introduction with more updated references related to the topic specially when it come to the references its just 23 references which not enough to express solid base to built on it.
2. In Results and Discussion: Authors should explain the difficulties they encountered in documenting from an accurate point of view as well as the errors in the pictures taken and how the pictures are connected. Also, they need to give an idea about the timeline of all this process.
3. Conclusions: because it is so brief, there is need to express a coherent conclusion.

By the end, I want to say that you did a great job and all this comments is to encourage the author to enhance the quality of paper.

 Thank you for your efforts.

 Best Wishes

Is the work clearly and accurately presented and does it cite the current literature?

Yes

If applicable, is the statistical analysis and its interpretation appropriate?

Yes

Are all the source data underlying the results available to ensure full reproducibility?

Yes

Is the study design appropriate and is the work technically sound?

Yes

Are the conclusions drawn adequately supported by the results?

Partly

Are sufficient details of methods and analysis provided to allow replication by others?

Yes

Reviewer Expertise:

BIM, Intelligent Buildings, Zero Energy, Smart Cities, Daylighting

I confirm that I have read this submission and believe that I have an appropriate level of expertise to confirm that it is of an acceptable scientific standard, however I have significant reservations, as outlined above.

# Response

**Published:** 2023-03-20

**Authors:** Sherif El Adawy

**Affiliations:** Arab Academy for Science Technology & Maritime Transport, Egypt

We would like to thank you, Dr. Osama Omar, for your comments.

 In replying to your comments, we took your comments into consideration for the new version of the article:

1. All 23 references covered throughout the article are mostly up-to-date in the research's field, and the use of hyperlinks has been recommended by the editor to help readers find up-to-date information on terminologies, organizations, platforms, laws, books, people, and tools. Moreover, we added various tools related to photogrammetry and digital archiving in the introductory section, which can be divided into 5 classes, and we selected DSLR cameras and smartphones that will be used in the case study for the documentation phase of HDPP.
2. In the “Results and Discussion” section, we clarified the difficulties we faced along the phases, which were concentrated at the first two phases, and also mentioned the phases' timeline for implementing the HDPP on the Société Immobilière building.
3. In the “Conclusions” section, we listed the advantages and disadvantages of what we learned from our experiences in this research and recommended solutions for the disadvantages that could be taken into consideration and used in the future.
